# Challenges in assessing the effects of environmental governance systems on conservation outcomes

**DOI:** 10.1111/cobi.14392

**Published:** 2024-10-17

**Authors:** Raphael A. Ayambire, Trina Rytwinski, Jessica J. Taylor, Matthew W. Luizza, Matthew J. Muir, Cynthia Cadet, Derek Armitage, Nathan J. Bennett, Jeremy Brooks, Samantha H. Cheng, Jenny Martinez, Meenakshi Nagendran, Siri Öckerman, Shannon N. Rivera, Anne Savage, David S. Wilkie, Steven J. Cooke, Joseph R. Bennett

**Affiliations:** ^1^ Department of City Planning University of Manitoba Winnipeg Manitoba Canada; ^2^ Canadian Centre for Evidence‐Based Conservation, Institute of Environmental and Interdisciplinary Science Carleton University Ottawa Ontario Canada; ^3^ International Affairs US Fish and Wildlife Service Falls Church Virginia USA; ^4^ School of Environment, Resources and Sustainability, Faculty of Environment University of Waterloo Waterloo Ontario Canada; ^5^ Global Science World Wildlife Fund Washington District of Columbia USA; ^6^ People and the Ocean Specialist Group, Commission on Environmental, Economic and Social Policy International Union for the Conservation of Nature Gland Switzerland; ^7^ Institute for the Oceans and Fisheries The University of British Columbia, AERL Vancouver British Columbia Canada; ^8^ School of Environment and Natural Resources Ohio State University Columbus Ohio USA; ^9^ Proyecto Titi, Inc. Orlando Florida USA; ^10^ Wildlife Conservation Society Bronx New York USA

**Keywords:** cogovernance, conservation actions, conservation interventions, evidence map, IPLC governance, shared governance, social outcomes, socioecological outcomes, acciones de conservación, cogestión, intervenciones de conservación, gestión compartida, gestión de IPLC, mapa de evidencias, resultados sociales, resultados socioecológicos

## Abstract

Effective governance is crucial for the success of conservation projects aimed at protecting wildlife populations and supporting human well‐being. However, few large‐scale, comprehensive syntheses have been conducted on the effects of different environmental governance types on conservation outcomes (i.e., biological and ecological effectiveness or effects of conservation on human well‐being), and clarity on the quantity and quality of evidence remains dispersed and ambiguous. We attempted a systematic map of the evidence on the effectiveness of different governance types to meet desired conservation outcomes in Africa, Asia, and Latin America. However, early in this effort, we observed a general lack of empirical research on the links between governance and conservation outcomes. To fill observed data gaps in the evidence base, we tried triangulating governance data from alternative sources (Protected Planet database) and pooling evidence from research conducted within the same conservation areas. Limited data were contained in the Protected Planet database, and governance types in conservation areas and landscapes were complex, making it difficult to use these approaches to assign governance types to conservation areas. To illustrate our observations from the failed systematic map attempt, we prepared a rapid evidence map that outlines a subset of the evidence base of articles linking governance types and governance principles with conservation outcomes. Only 3.2% (34 of 1067) of the articles we screened directly related conservation outcomes to governance type, and even fewer related governance principles to conservation outcomes. Based on our findings, we recommend improving the evidence base by supporting empirical research and increasing the availability and quality of governance data in freely accessible databases. These recommendations are critical for enhancing understanding of the role of governance in conservation projects and improving conservation outcomes.

## Abstract (es)

Retos para la evaluación de los efectos de los sistemas de gestión ambiental sobre los resultados de conservación

**Resumen:**

Una gestión eficaz es crucial para el éxito de los proyectos de conservación destinados a proteger las poblaciones de fauna y flora y apoyar el bienestar humano. Sin embargo, hay pocas síntesis exhaustivas a gran escala sobre los efectos de los distintos tipos de gestión ambiental en los resultados de conservación (es decir, la eficacia biológica y ecológica o los efectos de la conservación sobre el bienestar humano), y la claridad sobre la cantidad y calidad de las pruebas todavía está dispersa y ambigua. Intentamos elaborar un mapa sistemático con evidencias de la eficacia de los distintos tipos de gestión para logar los resultados de conservación deseados en África, Asia y América Latina. Sin embargo, al principio de este esfuerzo observamos una falta general de investigación empírica sobre los vínculos entre la gestión y los resultados de la conservación. Para cerrar las brechas observadas en la base de pruebas, intentamos triangular los datos sobre gobernanza procedentes de fuentes alternativas (base de datos Protected Planet) y agrupar las pruebas de las investigaciones realizadas en las mismas áreas de conservación. La base de datos Protected Planet tenía datos limitados y los tipos de gestión en las áreas de conservación y los paisajes eran complejos, lo que complicaba el uso de estos enfoques para asignar tipos de gestión a las áreas de conservación. Para ilustrar nuestras observaciones a partir del mapa sistemático fallido, preparamos un mapa rápido con evidencias que resumía un subconjunto de la base de pruebas de artículos que vinculan los tipos y los principios de gestión con los resultados de conservación. Sólo el 3.2% (34 de 1067) de los artículos analizados relacionaban directamente los resultados de conservación con el tipo de gestión; fueron menos los que relacionaban los principios de gestión con los resultados de conservación. Con base en nuestros hallazgos, recomendamos mejorar la base de pruebas con apoyo a la investigación empírica y aumentando la disponibilidad y la calidad de los datos sobre gestiones en bases de datos de libre acceso. Estas recomendaciones son fundamentales para comprender mejor el papel de la gestión en los proyectos de conservación y mejorar los resultados de la conservación.

## INTRODUCTION

Governance, the “conscious determination of action via the use of various forms of power” (Borrini‐Feyerabend & Hill, 2015), plays a vital role in supporting the effectiveness of conservation projects. In this context, *governance* refers to the processes by which groups decide what are acceptable uses and behaviors in terms of natural resource access and use in a given area. Governance can be differentiated from management, which is the resources, plans, and actions that ensure policies and operational decisions made through governance are implemented (Lockwood, 2010). Thus, governance and management are distinct in that the latter involves the implementation of rules and regulations defined by governors, who decide how natural resources in their jurisdiction can be used and are responsible for implementing natural resource access and use policies. A central objective of both environmental governance and management is to ensure that conservation actions achieve their intended outcomes for the conservation of biodiversity and the well‐being of people (CEBC, 2021).

Good governance is critical for the effectiveness of conservation projects because it creates conditions that increase project feasibility by improving the likelihood of project uptake and success (Kehoe et al., 2021); addresses value conflicts by resolving differences in opinions and beliefs about management and use of natural resources (Gooden & ‘t Sas‐Rolfes, 2020); and improves stakeholder perceptions of governance (Di Franco et al., 2020). These 3 functions of governance help steer conservation toward practices that are more just, equitable, representative, and legitimate, leading to increased societal support and respect for rules (Bennett et al., 2019; Eklund & Cabeza, 2017; Gooden & ‘t Sas‐Rolfes, 2020).

Governance is complex and depends on various factors, including, but not limited to, the following: type of governance, ranging from those led by Indigenous peoples and local communities (IPLCs) to those led by governments with various hybrids in between (Table 1) (Borrini‐Feyerabend & Hill, 2015); stakeholder (and rights holder) engagement, which considers the “quality of the totality of the interactions between those governing and those governed” (Kooiman & Bavinck, 2005, p. 19); and the qualitative application of commonly held governance principles, such as legitimacy, accountability, transparency, and participation (Battista et al., 2016) (Table 2). These factors can affect the biological and human well‐being outcomes of conservation projects (Macura et al., 2015).

**TABLE 1:** Environmental governance typology based on the primary decision‐making authority.

Type of governance^a^	Description
Public governance	Decision‐making authority rests exclusively with a government entity. Examples include exclusively government‐controlled terrestrial and marine protected areas.
Public–private governance	Decision‐making authority rests with a combination of government and a private entity (e.g., financial institutions, corporations, companies, groups of investors, individual landowners, nongovernmental organizations [NGOs], and civil society groups). For the purpose of this map, this governance type does not include interventions where decision‐making authority is delegated to community‐based institutions. Examples include certain public–private partnered terrestrial and marine protected areas and market‐based forestry interventions (e.g., certain logging operations).
Private governance	Decision‐making authority rests with a private entity (inclusive of financial institutions, corporations, companies, groups of investors, individual landowners, NGOs, and civil society groups) and can include either not‐for‐profit or for‐profit schemes. For the purpose of this map, this category does not consider community‐based institutions as private entities. Examples include private conservation areas and private game farming, wildlife ranching, trophy hunting, and hotel‐based marine reserves.
Public–community governance	Decision‐making authority rests with a government entity and Indigenous peoples and local communities and their organizations or representatives. Examples include certain types of comanagement agreements between governments and Indigenous peoples or local communities.
Private–community governance	Decision‐making authority rests with a private entity (inclusive of private sector financial institutions, corporations, companies, groups of investors, individual landowners, NGOs, and civil society groups) and Indigenous peoples and local communities and their organizations and representatives. Examples include privately managed tourism or hunting concessions on Indigenous peoples and local communities land, as well as certain community conservancy models.
Public–private–community governance	Decision‐making authority is shared among a plurality of entitled governmental and nongovernmental partners, private entities, and Indigenous peoples and local communities and their organizations and representatives. Examples include certain types of transboundary conservation areas, transfrontier conservation areas, and biosphere reserves.
Indigenous peoples and local communities governance	Decision‐making authority rests with Indigenous peoples or local communities. Examples include Indigenous peoples and local community conserved areas and territories.

^a^ Adapted from Lemos and Agrawal (2006), Borrini‐Feyerabend et al. (2013), and Baghai et al. (2018) and further refined here by splitting shared governance into narrower categories that provide more specificity on the nature of the collaborative arrangement (public–private, public–community, private–community, and public–private–community governance) for more clarity and nuance on governance types.

**TABLE 2:** A proposed^a^ set of principles for good governance.

Principle	Description
Fit	The intervention's design recognizes the scale of environmental threat, aligns governance activities between formal and informal institutions, and within the specific local socioeconomic and cultural context, and considers the elements of human well‐being as a long‐term goal of addressing the threat.
Legitimacy	The intervention possesses legal authority to make decisions and implement actions that are respected by stakeholders, including provision of secure land and natural resource tenure or both and adherence to legal obligations to rights holders.
Inclusivity	The intervention empowers equitable representation of and respect for the interests and priorities of stakeholders and rights holders most affected by the intervention, especially those from vulnerable and marginalized groups, and recognizes human and cultural rights.
Fairness	The intervention avoids bias in decision‐making and conflict resolution and distributes costs and benefits fairly.
Transparency	The intervention clearly and openly communicates rationale for decision‐making and makes information freely available and accessible.
Accountability	The intervention clearly articulates and assigns roles and responsibilities of all stakeholders to appropriate levels and holds all stakeholders to account; accountability is inclusive of horizontal (i.e., among stakeholders at the same governance level [e.g., within a local community]) and vertical accountability (i.e., upward and downward governance accountability across different levels [e.g., between the state, nonstate partners, and local community stakeholders]).
Support networks	The intervention possesses multilevel networks and relationships to promote coordination and collaboration within the engagement processes.
Capability	The intervention builds capacity and institutional leadership to promote compliance with, and enforcement of rules, and the ability to resolve conflict.
Knowledge coproduction	The intervention recognizes the contributions of both scientific and local and Indigenous knowledge, and stakeholders coproduce knowledge.
Resilience	The intervention possesses adaptive capacity to social, environmental, and climatic changes.

^a^ Proposed in CEBC (2021) and adapted from Bäckstrand (2006), Lockwood (2010), Lockwood et al. (2010), Biermann and Gupta (2011), Armitage et al. (2012, 2019, 2020), Plummer et al. (2013), Turner et al. (2014), Bennett and Satterfield (2018), Hare et al. (2018), van der Molen (2018), Béné (2020), Pomeranz and Stedman (2020), and Wilkie et al. (2015).

There is increased interest from conservation researchers, policy makers, and managers in understanding the linkages between governance‐related factors and conservation effectiveness. For example, Brooks et al. (2013) analyzed the synergies and trade‐offs among conservation outcomes and found that governance‐related factors such as capacity building, tenure regimes, and the cultural alignment of interventions are crucial for achieving joint success. Similarly, McKinnon et al. (2016) studied the impacts of conservation interventions on human well‐being and highlighted the need for further analysis and synthesis of governance factors that affect conservation–human well‐being linkages and outcomes—a recommendation that was echoed by Eales et al. (2021) in the context of marine conservation in Southeast Asia. Other reviews have stressed the importance of aligning community‐based conservation institutions with local socioecological conditions and recognizing power dynamics and networks (Fariss et al., 2023; Galvin et al., 2018).

Although the literature on the linkages between governance and conservation outcomes is expanding, there are still gaps in understanding. Several studies show inconsistencies in reporting of governance and community engagement approaches. For example, Macura et al. (2015) mapped evidence of the impact of governance type on the conservation effectiveness of forest‐protected areas and found that the evidence base is limited in terms of size, quality, and geographical area. Similarly, Raschke et al. (2019) examined the relationship among governance, community engagement, and outcomes of terrestrial conservation projects and concluded that the current evidence base is insufficient to enable the evaluation of the influence of governance and community engagement on conservation outcomes. Specifically, there was a “significant lack of coherence in the characterization of community engagement approaches, which impedes robust evaluation of utility and impact” (Raschke et al., 2019, p. 19). Moreover, a more nuanced understanding of the complex dynamics of governance is required to improve the biological and social effectiveness of conservation interventions (Armitage et al., 2020; Hajjar et al., 2020; Mahajan et al., 2021; Salerno et al., 2021). Further, recognizing gender as a critical element to understanding the dimensions of both governance and conservation practice is still an overlooked concept. For example, including women in resource management groups and conservation efforts can improve local natural resource governance (Westermann et al., 2005). Leisher et al. (2016) conducted a systematic map to provide an overview of the existing evidence linking gender composition of community groups managing natural resources to resource governance and conservation outcomes in forestry and fisheries and found that including women in resource management groups improved local natural resource governance in India and Nepal. However, they noted substantial gaps in the evidence base for other regions.

Furthermore, a recent crosswalk analysis of the International Union for Conservation of Nature (IUCN) Green List of Protected and Conserved Areas and protected area effectiveness assessment methods highlights persistent gaps in protected area management effectiveness assessments, including limited efforts to measure whether a site is effectively or equitably governed (UNEP‐WCMC & IUCN, 2022, p. 7). Consequently, research advancing governance effectiveness in conservation is needed.

In line with this need, the International Affairs Program of the United States Fish and Wildlife Service (USFWS‐IA) commissioned a systematic map to provide an overview of the literature on the effectiveness of community‐based conservation (subsequently expanded to Indigenous peoples and local community governance [IPLC]) relative to other types of governance for species of conservation concern and human well‐being outcomes. The general aim of a systematic map is not to provide an answer to a question about the impact of an exposure or the effectiveness of an intervention or test a hypothesis. Instead, it is to provide a description of what research has been undertaken and to identify knowledge gaps (subtopics requiring additional primary research) and clusters (subsets of evidence that may be suitable for secondary research) in the evidence base (Haddaway et al., 2016; James et al., 2016). While developing the systematic map, we discovered that the evidence base on the effectiveness of governance on both biological and human well‐being conservation outcomes was limited and multiple challenges were encountered. Here, we critically reflected on these data gaps and challenges and devised recommendations for future research and policy to improve the evidence base on governance and conservation effectiveness. Specifically, we first outlined the research context and described the methods used for our attempted systematic map concerning the effectiveness of environmental governance, with a special emphasis on species of conservation concern. Related to the research context, we outlined a typology for assessing governance effectiveness by suggesting the splitting of the IUCN shared governance type. Next, we described the challenges encountered while attempting to triangulate governance data from key sources, highlighting their limitations in facilitating governance effectiveness assessment. We then described the methods used and presented the results of a rapid evidence map that outlines a subset of the evidence base of articles linking governance types and principles with conservation outcomes. Finally, we provided practical recommendations for improving research and policy aimed at enhancing the evidence base on environmental governance effectiveness.

## METHODS

### Study context

The USFWS‐IA has a strong interest in community‐based conservation projects, spanning its global programing support for wildlife conservation. In 2019, USFWS‐IA conducted a comprehensive portfolio analysis of its funding for community‐based conservation projects in sub‐Saharan Africa, which revealed the importance of such initiatives in their investments in Africa (Luizza & Gorenberg, 2019). However, uncertainty remained regarding community‐based conservation project effectiveness and impact compared with other conservation approaches for the species targeted by USFWS international programing. Consequently, USFWS‐IA commissioned an evidence synthesis project led by the Canadian Centre for Evidence‐Based Conservation (CEBC) to assess how the ecological effectiveness and social impacts of conservation vary across governance types, with an emphasis on community‐based conservation projects in Africa, Asia, and Latin America. The intention was that results of the evidence synthesis would support evidence‐based program decisions to ultimately improve the impact of conservation efforts supported by USFWS‐IA financial assistance programs.

In addition to the core team from CEBC and USFWS‐IA, an advisory team (which included all authors of this article) was established and consulted in the design of this systematic map protocol. This included the iterative and collaborative formulation of the question and its components, the search strategy, and eligibility criteria. Advisory team members represented a broad range of scholars and practitioners with research interests in and practical expertise with both the natural and social sciences and a diverse geographic scope. More specifically, members were selected based on specific knowledge and experience related to conservation, governance, and human well‐being dimensions of conservation within the 3 regions of Africa, Asia, and Latin America.

**FIGURE 1:**
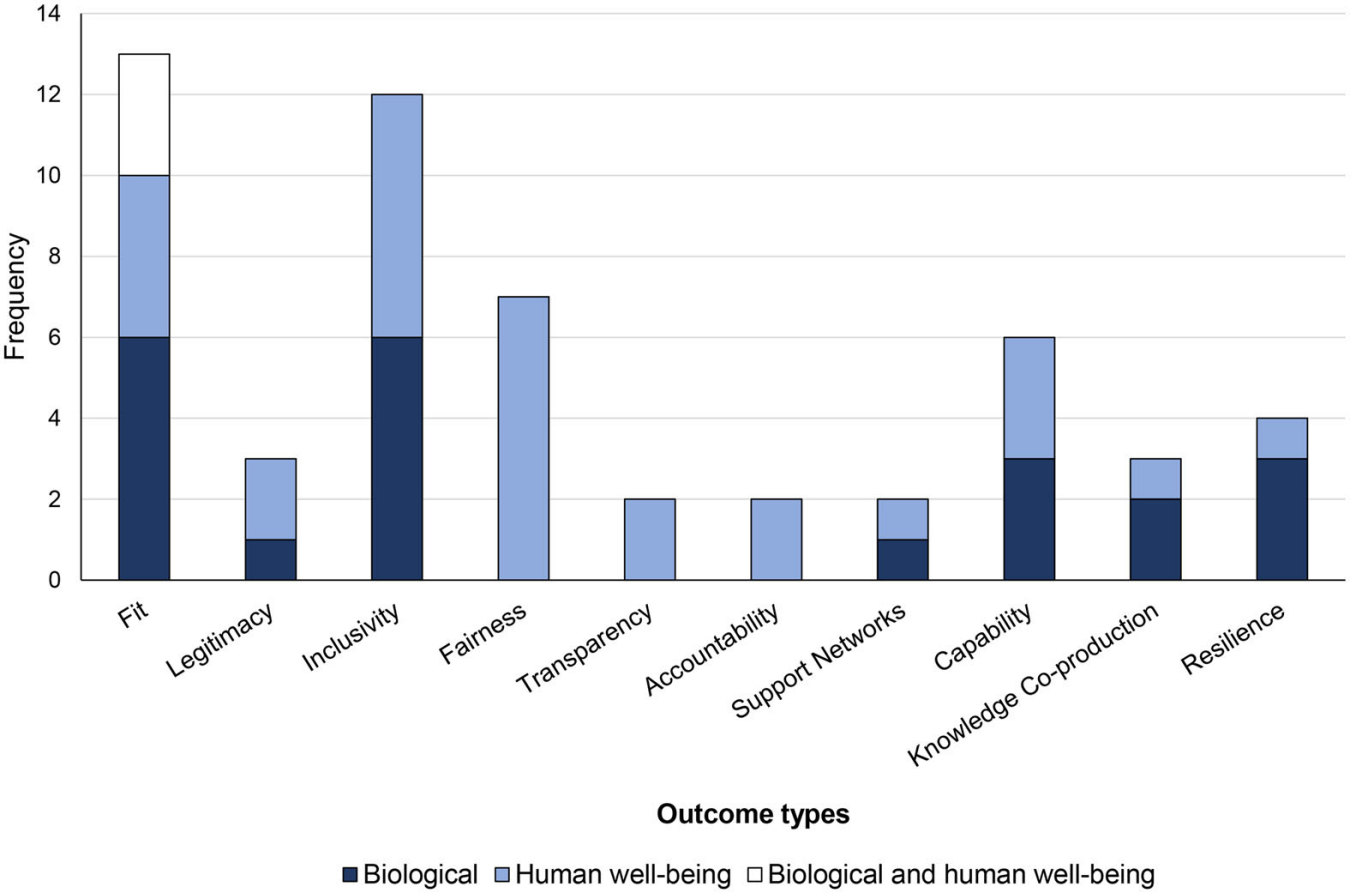
Frequency of governance principles identified as being present or absent from 33 out of the 34 articles linking governance and conservation outcomes (governance principles defined in Table 2).

With the support of an advisory team, governance types were defined and refined, with the focus on clarifying and specifying the diversity of IPLC involvement in governance (Table 1). Specifically, the shared governance type in the IUCN typology (Borrini‐Feyerabend et al., 2013) was split into 4 narrower categories that specified the nature of the collaborative governance type: public–private, public–community, private–community, and public–private–community governance (Table 1). This categorization built on the environmental governance typology developed by Lemos and Agrawal (2006). These narrower categories allowed for a more nuanced analysis of the different types of institutional arrangements, partnerships, and collaborations that IPLCs engage in with other actors, such as governments or nongovernmental organizations. They also reflect the varying degrees of power and influence that IPLCs have in decision‐making processes (Simkins et al., 2024). A framework was developed to conceptualize the focus of the systematic map, which portrays the interaction between an intervention's governance approach and types and its outcomes (see figure 1 in the systematic map protocol [CEBC, 2021] for further details). We acknowledge that the effectiveness of governance types can be influenced by the levels of stakeholder (and rights holder) engagement and, subsequently, the presence or absence of good governance principles (Table 2).

### Development of a systematic map

We initiated (but did not complete) a systematic map to collate and summarize the evidence on the effectiveness of environmental governance types (Table 1) related to selected focal species and multidimensional domains of human well‐being in Africa, Asia, and Latin America. Components of the systematic map are in Table 3, and screening and study inclusion criteria are in section 3.2 of CEBC (2021). We were also interested in summarizing the available information on the presence and absence of commonly cited good governance principles (Table 2) and the presence and absence of community engagement (see figure 2 in the original systematic map protocol [CEBC, 2021]). This mapping exercise was based on searches conducted in 2021 (i.e., commercially published and gray literature) of 4 bibliographic databases (i.e., Web of Science Core Collection, Scopus, ProQuest Dissertations & Theses Global, and Science.gov) accessed from Carleton University's institutional subscriptions and one online search engine (Google Scholar). English search terms were used to conduct all of our searches (Table 4). We also hand‐searched the bibliographies of 149 relevant reviews identified from the searches above to evaluate relevant titles that may not have been found using the search strategy. Additionally, we issued a call for evidence to target sources of gray literature through relevant mailing lists, social media, and distribution to relevant networks and colleagues by the advisory team. This yielded 140,799 unique records after duplicate removal for title and abstract screening (details on all information sources and search numbers are in Appendix S1).

**TABLE 3:** The PICO (population, intervention, comparator, outcome) components of the original systematic map question (from CEBC [2021]) used to screen for study eligibility for the original systematic and rapid evidence maps of the evidence on the effectiveness of different governance types to meet desired conservation outcomes in Africa, Asia, and Latin America.

Population	Intervention	Comparator	Outcome
Species and species groups targeted by International Affairs Program of the US Fish and Wildlife Service from Africa, Asia, and Latin America (list in Appendix S2) or Indigenous peoples and local communities associated with a conservation intervention in Africa, Asia, and Latin America (or both)	Various types of governance (Table 1)	No studies excluded based on the presence or absence of a comparator^a^	Measures of change in focus species biological outcomes, social outcomes, and multidimensional domains of human well‐being

^a^ Although a comparator was not required for inclusion for the title and abstract screening for the original systematic map, it was required when screening for the rapid evidence map (i.e., only articles that provided a direct link between governance type and an eligible conservation outcome, including a comparator [i.e., a temporal comparison: before intervention or spatial comparison, or both; no intervention or comparison between different types or levels of intervention but all types of governance needed to be known], were included [details in Appendix S3]).

**TABLE 4:** Search string (optimized for Web of Science core collections) for the execution of the searches to locate commercially published and gray literature for the systematic map of the evidence on the effectiveness of different governance types to meet desired conservation outcomes in Africa, Asia, and Latin America.

Component	Search string
Population	TS = ((Wildlife OR Fauna OR Animal$ OR Mammal$ OR ((Endangered OR Threatened OR vulnerable) NEAR/3 species) OR Elephant* OR Rhino* OR Antelope$ OR Gazell* OR Tiger$ OR Lion$ OR Panther$ OR Leopard$ OR Cheetah$ OR Ocelot$ OR Jaguar$ OR Pangolin$ OR Anteater$ OR “Ant eater$” OR Giraff* OR Okapi$ OR Primate$ OR Ape OR Apes OR Gorilla$ OR Chimpanzee$ OR Orangutan$ OR Gibbon$ OR Parrot$ OR Macaw$ OR Turtle$ OR Tortoise$ OR Cyca* OR Ivory OR Bushmeat$ OR Buffalo* OR flora) NOT (Rhinovirus* OR Rhinoplast*))
	AND
Intervention	TS = (“protected area$” OR (reserve$ NEAR/3 (natur* OR forest OR wildlife OR game OR private OR biosphere OR special)) OR “key biodiversity area$” OR “national park$” OR “wildlife sanctuar*” OR “wildlife refuge$” OR “wilderness area$” OR “marine protected area$” OR “MPA$” OR “private governance” OR “game farming” OR “wildlife ranching” OR “trophy hunting” OR “Community Resource Management Area$” OR “Wildlife Manage*” OR (“community based” NEAR/3 conservation) OR “CBC” OR (“community based” NEAR/3 management) OR “CBNRM” OR “community managed” OR ”community based governance” OR “collaborative management” OR (collaborative NEAR/3 governance) OR “collaborative management” OR “co‐management” OR “comanagement” OR “environmental stewardship” OR “Wildlife Manage*” OR “wildlife governance” OR “community forest*” OR “Forest* Manage*” OR “Fisher* Manage*” OR “small scale” OR “Payment For Ecosystem Service$” OR “ecotourism” OR “Indigenous Peoples’ and Community Conserved Territories and Area$” OR “ICCA$” OR “Indigenous Protected Areas” OR “Locally Managed Marine Area$” OR “Indigenous” OR Aboriginal* OR “Native peoples” OR Tribal OR Tribe$ OR (conserv* NEAR/3 (governance OR area OR “community‐led” OR private OR communit* OR status OR designation OR strateg* OR assessment$ OR policy OR policies OR significance OR action$ OR activit* OR manage* OR conservanc* OR covenant$ OR concession$ OR easement$ OR plan* OR priorit* OR decision)))
	AND
Outcome	TS = (“Population” OR “Relative size” OR Abundance$ OR Densit* OR Biomass OR Status OR Presence$ OR Distribution OR Range$ OR Occupanc* OR Detect* OR Recovery OR Progress OR Protect* OR Reproducti* OR Migration OR Behavior$ OR Behaviour$ OR “Genetic diversi*” OR Fecundity OR “Age structure” OR “Size structure” OR Recruitment OR “Biotic response” OR “Biological response” OR “Conservation target” OR Biodiversity OR “Ecological response” OR Impact OR Effectiveness OR Effective OR “outcome$” OR “social outcome$” OR “social capital” OR “social impact$” OR “social justice” OR “socially just” OR “well‐being” OR “well‐being” OR awareness OR adoption OR “willingness to” OR welfare OR security OR livelihood OR job OR employment OR asset OR income OR decision‐making OR govern* OR empower* OR participat* OR equity OR “human health” OR nutrition OR mortality OR disease OR consumption OR skill* OR degree OR train* OR literacy OR access OR “water clarity” OR “water quality” OR “clean water” OR “food security” OR vulnerability OR attitude* OR perception* OR “human capital” OR sanitation OR “building materials” OR housing OR fuel OR expenditure OR safety OR adapt* OR resilien* OR efficien* OR coproduction OR capability OR consensus OR integration)

We used a semiautomated approach for title and abstract screening by employing a text‐based machine learning algorithm in the EPPI‐Reviewer Web software (https://eppi.ioe.ac.uk/EPPIReviewer‐Web/home) to prioritize relevant articles (Thomas, 2013). The EPPI‐Reviewer's priority screening effectively reduces the screening burden by up to 60% while retaining human involvement and control over the screening process and outcomes (Tsou et al., 2020). The EPPI‐Reviewer is one of the most commonly used tools in environmental management and conservation evidence syntheses for the Collaboration for Environmental Evidence (CEE, 2022) and a recommended tool of Cochrane Reviews in the healthcare field (Lefebvre et al., 2023).

Prior to screening titles and abstracts, we performed a consistency check to ensure consistent and repeatable decisions were being made by reviewers. This included 3 reviewers independently screening a random subset of 1000 titles and abstracts. We then performed a comparison of the consistency check items, discussing any disagreements among reviewers. Agreement was tested formally with a kappa test (Cohen, 1960). Reviewers obtained a score of ≥0.6, indicating a high level of consistency. We then used this unbiased subset of articles from the consistency check as the training set for the machine learning algorithm in EPPI‐Reviewer, after which screening continued with a single reviewer screening each article. During this priority screening, we identified a logical cutoff point (i.e., a plateau where new articles were no longer being included) at which title and abstract screening was stopped. This plateau occurred after screening titles and abstracts of 14,387 (10.2%) articles. Of these 14,387 articles, 4417 (30.7%) met our inclusion criteria (Table 3 and section 3.2 in CEBC [2021]) and were included in our analysis. Only English‐language literature was screened. More articles likely exist in other languages; however, we did not have the resources to conduct these searches.

When screening the titles and abstracts of the 14,387 articles, we took an inclusive approach, considering that most articles lacked clear justification for exclusion based on the limited information provided in the titles and abstracts. However, on closer examination of the included articles at the full‐text screening stage of our attempted systematic map, we found that many of them did not provide a direct link between governance and conservation outcomes. So that we could use the large data set, we triangulated data from alternative sources. This was done in part to enable us to achieve our study objectives for the systematic map but also to generate new insights to guide future efforts.

### Triangulating governance data

We used the Protected Planet database (https://www.protectedplanet.net/) to triangulate available governance data with articles that reported conservation outcomes in specific areas or landscapes. We chose the Protected Planet database because it is the most comprehensive and updated source of information on governance and management authority of protected and conserved areas (including other effective area‐based conservation measures [OECMs]). For instance, studies that reported conservation outcomes in the Ruaha National Park in Tanzania (e.g., Abade et al., 2018) can be associated with “public governance” or “federal or national ministry or agency” governance type as specified in the Protected Planet database. Similarly, studies reporting conservation outcomes in Tanzania's Pawaga‐Idodi Wildlife Management Area (e.g., Kiwango et al., 2018) can be associated with “local communities” or “Indigenous peoples & local communities governance.” This approach was effective for papers that specified locations or used names that matched those reported in the database (Abade et al., 2018; Goossens et al., 2016; Kiwango et al., 2018).

However, we encountered challenges when using the Protected Planet database to assign governance types to conservation areas in several of our sample articles. For example, some articles used names that did not match those in the database; had study areas not captured in the database; or had study areas included in the database, but the governance designation was not reported. Yang and Xu (2003), for instance, studied the biological and ecological diversity of the Changbai Mountain Biosphere Reserve (i.e., Changbaishan in the Protected Planet database) in China. Although this Biosphere Reserve is in the Protected Planet database, information on the governance type is not provided. Several similar examples were discovered. Indeed, at the time of writing, there were approximately 18,732 protected areas and OECMs in the database without their governance type provided, which limited the utility of the database for our study. We speculate further that even if more details on governance type were included in the Protected Planet database, there would likely be differences in how those types were categorized.

Another challenge was the mismatch between the governance and spatial scales of study locations. For example, some studies were conducted in landscapes spanning multiple governance types, such as the Ruaha landscape in Tanzania, which includes the Ruaha National Park, game reserves, village lands, and community‐led wildlife management areas (Kent & Dickman, 2022). Environmental governance in such transboundary landscapes is inherently multiscalar, with governance types nested across levels from local to national. However, the Protected Planet database captures only the Ruaha National Park, whereas some empirical studies span the entire landscape (Abade et al., 2018). Similarly, the Kavango‐Zambezi Transfrontier Conservation Area (KAZA TFCA) spans 5 southern African countries, encompassing “a mosaic of land cover and governance types, including national parks, urban areas, communal grazing and farmlands, and community‐based conservation areas” (Drake et al., 2020, p. 3). Though individual studies in KAZA may focus on specific locations, they often do not specify which precise parts or scales of governance they analyzed. This lack of clarity about the scale and context of governance in study methods prevents accurate categorization and linkage to databases like Protected Planet.

Also, we identified clusters of the evidence based on geographic or area‐based criteria and compiled them into an evidence pool. Then, we used the data from this pool to augment individual papers and address the gaps in the governance data. Our objective was to establish links between articles reporting conservation outcomes and the specific types of governance prevalent in the study regions. This approach was founded on the premise that conservation actions do not operate independently of the wider governance context in which they are implemented (Brooks et al., 2012). Through this analysis, we aimed to infer whether better or worse conservation outcomes are associated with particular types of governance. For example, in the KAZA TFCA, we found at least 14 articles, of which only 5 contained data on both governance and conservation outcomes. The remaining 9 articles only reported on conservation outcomes. Our plan was to use the pool of governance data from the 5 articles to establish links with conservation outcomes reported in the other 9 articles. However, our attempt at evidence pooling did not work because of the mismatch between the governance scales and spatial scales of the different study locations, as discussed above.

### Development of a rapid evidence map

Following the failed attempts to identify sufficient evidence for the systematic map, we decided to share our experience and call for more empirical research on the influence of environmental governance on biological and human well‐being outcomes. To illustrate our observations, we prepared a rapid evidence map. The aim of the rapid evidence map and the systematic map was the same—they are tools for describing existing evidence on a broad topic or policy domain that provide a snapshot of the current state of knowledge and are designed to highlight both evidence clusters and gaps. We refer to this exercise as a rapid evidence map because we have taken a demonstrative sample of the evidence base identified from our systematic map searching and screening efforts. Our intent with this rapid evidence map was to provide support for our observations that there was indeed limited evidence linking environmental governance and biological and human well‐being outcomes, rather than undertaking a more comprehensive assessment of the current state of knowledge, which was not feasible due to logistical constraints.

For the rapid evidence map, we randomly selected a representative subset of 1104 articles (25% of the original 4417 articles included at the title and abstract screening stage of the systematic map). Random selection of articles was carried out by assigning a random number to each of the 4417 articles with the RAND() function in Excel and then sorting the list of articles by their random numbers. We then selected the first 1104 articles from the list and evaluated them further, focusing on 2 main questions: what proportion of articles provide a direct empirical link between governance type and biological or human well‐being or both outcomes in relation to conserving species targeted by USFWS‐IA grant programs or to Indigenous peoples or local communities in Africa, Asia, or Latin America and of the articles identified through the former question, what proportion provides sufficient information (in the abstract, full‐text, or both) to identify the presence or absence of good governance principles, as presented in Table 2?

We focused on understanding the quantity of evidence that directly links governance and conservation outcomes; thus, we present our results in a simple matrix indicating the frequencies of articles that fit a stricter inclusion criterion delineated by our main research questions. Specifically, we included articles that met the following criteria. First, there had to be an empirical (qualitative, quantitative, or both) evaluation of the effectiveness of an environmental governance type or types on biological or human well‐being or both outcomes based on a comparative approach, that is, a comparison of the intervention (governance type) with a counterfactual in the form of a time period, location, or control group, or all 3 in the absence of the intervention or different intervention types or levels of the same intervention. Second, it had to be a primary study. Finally, the species or species group or groups had to be native to Africa, Asia, or Latin America and targeted by USFWS international activities or pertain to Indigenous peoples or local communities associated with a conservation intervention located in the 3 relevant regions, or both (see Appendix S3 for details of rapid evidence map inclusion criteria).

We only evaluated articles for inclusion in the rapid evidence map at the title and abstract screening level under the assumption that if one of the objectives of the article was to evaluate the effectiveness of at least one governance type on biological or human well‐being or both outcomes, such an empirical testing would be mentioned or described in the abstract. Therefore, if an article did not contain an abstract, it was excluded from consideration in the rapid evidence map. This was the case for 37 of the 1104 subset of articles, leaving 1067 articles for analysis in the rapid evidence map. It is unclear how many of these articles (if any) would have met all the inclusion criteria. Including a larger subset of articles would have added strength and accuracy to our rapid evidence map.

## RESULTS

The rapid evidence map showed that only 3.2% of articles (*n* = 34/1067) directly investigated (i.e., explicitly evaluated qualitatively, quantitatively, or both) environmental governance types and reported information on biological outcomes (*n* = 18), human well‐being outcomes (*n* = 13), or both (*n* = 3) (Table 5). Most of these articles focused on cases from sub‐Saharan Africa (*n* = 22), followed by Asia (*n* = 9) and Latin America (*n* = 3). Examples of these articles included Hardouin et al. (2021), in which the authors investigated how public and IPLC governance types influenced the population density of 3 lesser‐studied carnivores in different habitats in Tanzania; Bajracharya et al. (2005), in which the authors examined the effectiveness of community‐based approaches for conservation of biodiversity in Annapurna Conservation Area (Nepal) through ecological and social assessments and identified the factors that contributed to the positive outcomes as well as the challenges posed by political instability; and Miorando et al. (2013), in which the authors compared the abundance of *Podocnemis sextuberculata*, a vulnerable turtle species, in the lower Amazon between neighboring areas with and without community‐based management initiatives and found that the former had higher turtle populations influenced by environmental variables. Of these articles, 97.1% (*n* = 33/34) provided information on the presence or absence of governance principles when directly linking governance and a biological outcome (*n* = 17), a human well‐being outcome (*n* = 13), or both outcome types (*n* = 3) (Table 5). These numbers indicated a limited evidence base for linkages between governance and conservation outcomes. For those few articles that examined these linkages, almost all provided sufficient information to identify the presence or absence of governance principles (Figure 1). When we further refined our sample by removing articles that did not clearly focus on eligible species or species groups (Appendix S2), the number of articles providing a direct link between governance and conservation outcomes dropped to 1.1% (*n* = 12/1067) (Table 5). Notably, all 12 articles addressed linkages between governance types and biological outcomes. This finding highlights a critical gap in the available evidence base, as no articles in our sample investigated the links between governance and conservation outcomes for our eligible species in the domain of human well‐being outcomes. A full list of articles used in the rapid evidence map and their screening results is in Appendix S4.

**TABLE 5:** Gap matrix describing articles linking governance and conservation outcomes.

	Direct link with an environmental governance type	Sufficient information to identify presence/absence of governance principles when a direct link is provided
Biological outcomes	18 (12)	17 (11)
Human well‐being outcomes	13 (0)	13 (0)
Biological and human well‐being	3 (0)	3 (0)
% of (subset) evidence base	3.2 (1.1)	97.1 (91.7)

*Note*: Values in parentheses are results from further refinements to our sample by removing articles that did not clearly focus on eligible species or species groups (Appendix S2) or did not specify in the abstract the species investigated.

## DISCUSSION

We set out to examine the evidence base linking governance and conservation outcomes. We found that only 3.2% of the articles we screened directly related conservation outcomes to governance type, and even fewer articles related governance principles to conservation outcomes (Table 5). These findings are consistent with Macura et al.’s (2015) and Zhang et al.’s (2023) observation that published research on the linkages between governance and conservation effectiveness is limited. Although we did not explore the limitations in quality, as noted by Macura et al. (2015), our results indicated that the evidence base may also be limited in terms of species‐specific conservation effectiveness. Additionally, the small sample of evidence assessing species‐specific conservation effectiveness appeared to focus only on biological outcomes, suggesting no emphasis on human well‐being outcomes.

We also developed and tested 2 approaches to fill the data gaps. Overall, our findings suggest that the evidence base is limited and that utilizing complementary data sources to supplement the evidence base entailed challenges. These findings highlight a critical gap in the literature and the need for more robust and coherent empirical research on governance and conservation outcomes.

We recommend the following to improve the evidence base of governance and conservation effectiveness: provide support for empirical research on the influence of governance on conservation outcomes and increase access to readily available governance data to complement field‐based empirical data (summarized in Table 6).

**TABLE 6:** Summary of gaps in the current evidence base linking governance and conservation outcomes and recommendations for improving this evidence base.

Gap	Recommendation or agenda	Who can help
Lack of empirical research linking governance and conservation outcomes	Enhance investments in empirical research on the influence of environmental governance on conservation outcomes, especially in the human well‐being outcomes domain (in addition to focusing on ecological and management studies).	Funders
	Encourage interdisciplinary and transdisciplinary conservation research. Conduct high‐quality empirical research on conservation outcomes with governance as an analytical focus, including a focus on understanding how key characteristics of particular governance modes and models (e.g., adaptive governance, anticipatory governance, etc.) influence outcomes. Test and refine frameworks and codevelop indicators for measuring the influence of governance on conservation outcomes.	Funders Researchers Communities
Inadequate systematic reporting of governance data	Establish and utilize consistent dimensions of governance.	Researchers Communities International conservation organizations
Limited accessibility of governance data	Enhance the accessibility of governance data by improving online databases and tools.	Researchers International conservation organizations

### Empirical research on the influence of governance on conservation outcomes

Adequate and high‐quality scientific evidence is crucial for good environmental decision‐making (Bennett, 2022). However, as our evidence map demonstrates, empirical research on the influence of governance on conservation outcomes is limited. This dearth of evidence underscores the critical need for researchers and funders to enhance the rigor (using appropriate comparators to establish strong causal relationships) (Christie et al., 2020) and availability of empirical research on the influence of governance on conservation effectiveness. Given the multifaceted nature of governance, understanding its influence on conservation outcomes necessitates an analysis of several dimensions, as outlined in Table 7. Likewise, because both the biological and human well‐being domains are central to successful conservation, it is imperative that understanding be broadened by conducting more empirical research focused on the aspects of human well‐being (Ban et al., 2019; Milner‐Gulland et al., 2014), an area that has been relatively overlooked, especially pertaining to research targeting species of global conservation concern, as outlined in Table 5.

**TABLE 7:** Dimensions of governance with potential influence on conservation outcomes.

Governance dimension	Description and potential influence on conservation outcomes
Stakeholder or rights holder engagement	Involvement of different actors in governance of natural resources, such as policy makers, implementers, beneficiaries, community members, and civil society groups; often a legal requirement and can enhance the legitimacy, accountability, and effectiveness of conservation decisions and actions (Armitage et al., 2012)
Property rights systems (tenure)	Regulations dictating who has access to and control over conservation resources, including private, state, community, open access, or hybrid property regimes that establish rights related to withdrawal, management, exclusion, and alienation (Vincent, 2015); property‐rights systems can influence the incentives, behaviors, and outcomes of resource users and managers
Scales at which governance operates	Spatial, temporal, and institutional levels at which governance processes take place, such as local, national, regional, or global; coordination, coherence, and responsiveness of governance to the socioecological context of conservation can be affected by scale (Cash et al., 2006)
Governance types	Modes or mechanisms of governance that shape how decisions are made and implemented, such as private governance, public governance, Indigenous peoples and community‐based governance, and other hybrid modes of governance (Armitage et al., 2012) (Table 1); different values, norms, and interests of stakeholders can be reflected by governance types and influence the distribution of costs and benefits of conservation (Lemos & Agrawal, 2006)
Governance principles	Characteristics or principles that characterize good governance, such as transparency, inclusiveness, rule of law, legitimacy, accountability, fairness, and capability; trust and compliance of stakeholders can be affected by governance principles and contribute to the social and environmental outcomes of conservation (Armitage et al., 2020; Lockwood, 2010) (Table 2)
Free prior and informed consent (FPIC)	Recognizes the right of Indigenous peoples and local communities to give or withhold their consent to activities that affect their lands, territories, and resources; can ensure respect for the rights, cultures, and livelihoods of marginalized groups and foster more inclusive and sustainable conservation practices (Mahanty & McDermott, 2013)
Governance interplay	Captures interactions and linkages among governance actors, institutions, and processes across scales and sectors; governance interplay can create synergies or conflicts among different governance objectives and influence coherence and effectiveness of conservation policies and actions (Young, 2002)
Governance context	Considers broader socioecological factors that shape and are shaped by governance processes, such as culture, history, politics, economy, demography, and biogeography; governance context can provide opportunities or constraints for conservation governance and require adaptive and flexible approaches (Adger et al., 2003; Ayambire et al., 2022)

Additionally, developing frameworks and indicators to measure the influence of governance on conservation outcomes is necessary to increase the quality and quantity of evidence on the subject. Frameworks and indicators help to standardize measurement across different contexts and enable comparisons between studies. This is critical for building a robust evidence base to inform policy decisions regarding the most effective governance strategies for achieving conservation outcomes. Although there is certainly no shortage of governance frameworks and indicators (e.g., Bennett & Satterfield, 2018; Lockwood, 2010; Springer et al., 2021), they have not been widely and empirically tested and operationalized for measuring governance impacts on conservation outcomes. In advancing the evidence base on governance impacts on conservation effectiveness, it is essential for future research to focus on rigorously testing and refining existing frameworks through empirical studies in different contexts.

There is also the need for a shift from treating indicators as “technical, bureaucratic, and scientific challenge[s], … external from everyday politics and dynamics of social power” to one “that weaves power and knowledge together in the context of indicator development and implementation” (Muhl et al., 2022, p. 448). Codeveloping indicators with affected communities and other stakeholders and rights holders will be crucial to ensuring the appropriate indicators are devised (Sigouin et al., 2023). This process acknowledges the need for site‐specific indicators, developed by multiple stakeholders, that can assess the current state of and change over time in the status of good governance principles and conservation priorities. Several tools, such as the IIED's assessing governance at protected and conserved areas (GAPA) (https://www.iied.org/assessing‐governance‐protected‐conserved‐areas‐gapa), site‐level assessment of governance and equity (SAGE) assessment tools (https://www.iied.org/site‐level‐assessment‐governance‐equity‐sage), and the Elinor tool and data system (https://elinordata.org/), offer guidance on participatory methods. However, implementing these participatory approaches poses practical challenges, such as how to select a list of indicators that can capture the complexity and diversity of conservation outcomes and stakeholder perspectives and power dynamics.

Fortunately, promising examples of participatory indicator development and implementation are available to help address these challenges (Danielsen et al., 2009; Reed et al., 2008). For example, Zuniga‐Teran et al. (2022) propose an iterative and participatory process that involves regular consensus and review among stakeholders, as well as the use of multiple data sources and methods to measure and interpret the indicators. Their approach involved the use of stakeholder workshops and surveys to derive and rank indicators and the continuous tracking and modifications of indicators in relation to changes in conservation priorities. Additionally, in line with the insights from Laituri et al. (2023), we emphasize the need for reflective inquiry with communities spanning phases of engagement (i.e., before, during, and after onsite engagement). Facilitators of these participatory approaches hold a lot of power in determining what voices are heard, and this responsibility should not be taken lightly. Furthermore, governance and conservation research must be interdisciplinary and transdisciplinary, involving scholars and practitioners from diverse fields such as ecology, sociology, anthropology, economics, and political science. Interdisciplinary research (Lélé & Norgaard, 2005) enables a comprehensive understanding of the relationships between governance and conservation outcomes, recognizing that conservation issues are embedded in complex socioecological systems. Transdisciplinary research facilitates collaborations between researchers, practitioners, and decision makers and can foster mutual learning and coproduction of knowledge that is both scientifically rigorous and socially relevant (Mattor et al., 2014). By integrating interdisciplinary and transdisciplinary approaches, research on governance and conservation can foster the development of more consistent, widely accepted definitions, frameworks, and measurement indicators, providing a richer and more nuanced understanding of the influence of governance on conservation outcomes. Although researchers should play a pivotal role in addressing the research gaps, funding organizations must also prioritize increasing investments in research that evaluates the influence of governance on conservation outcomes and facilitate the formation and operation of interdisciplinary and multidisciplinary scientific communities and teams (Mattor et al., 2014). Moreover, IPLC stakeholders and rights holders in these conservation landscapes must be included at all stages of this work, with added and intentional consideration of processes to enhance participation and foster reflective inquiry to ultimately improve collaboration and equitable outcomes (Laituri et al., 2023).

### Access to readily available governance data to complement field‐based empirical data

We demonstrated the potential of utilizing available and easily accessible governance data to complement field‐based empirical research. However, we also found the need for more robust tools to capture data on the multiple dimensions of governance and conservation outcomes. Fortunately, several governance‐related tools and databases are being developed and scaled up globally to track key governance dimensions. Some of these tools and databases include the Protected Planet database (https://www.protectedplanet.net/), the IUCN Natural Resource Governance Framework (https://portals.iucn.org/library/node/49703), the IIED Governance Assessment and SAGE Assessment Tools, and the Elinor tool and data system (https://www.elinordata.org) (Mahajan et al., 2024). These tools aim to gather data within a standardized framework to enable comparisons of governance shifts over time and potentially over different sites to inform the assessment of the impacts of governance on conservation outcomes and guide conservation decision‐making by policy makers, practitioners, and communities.

To make these tools more useful for evaluating governance and conservation outcomes, it is necessary to increase the data coverage to address the various dimensions of governance and conservation outcomes. This requires increased engagement and alignment of efforts among researchers, conservation managers, conservation governors, and communities to identify and provide data needs. Additionally, efforts should be made to improve data sharing and accessibility to ensure that these valuable tools can be effectively utilized by a wide range of stakeholders. Increasing access to readily available governance data will contribute to a better understanding of the influence of governance on conservation outcomes and will enable more evidence‐based decision‐making for conservation practice. However, given that governance systems can be dynamic, conservation researchers should be cautious when pairing data from different sources and over different time periods to ensure that information on governance and on outcomes is consistent and up to date. Furthermore, when working with Indigenous peoples, conservation researchers must endeavor to respect Indigenous data sovereignty and other community‐based data sharing protocols that may operate outside of the standard data sharing agreements.

### Map limitations and broader implications

Our assessment of this evidence gap is based on an English‐language‐only evidence base for this topic. Therefore, although its purpose was to be demonstrative, this rapid evidence map cannot be considered exhaustive. The ability to conduct searches in other languages and to assess the entirety of the evidence base would add strength to the accuracy of this evidence map. Nevertheless, we posit that the knowledge gap identified from this illustrative exercise reflects a true knowledge gap and underscores the need for more and timely empirical research on the influence of governance on conservation effectiveness. In addressing this need, it is important to broaden the scope of governance research related to conservation and facilitate greater consistency in frameworks and indicators for measuring the impact of governance on conservation effectiveness.

Despite the growing awareness of the importance of understanding the influence of governance on conservation effectiveness, our rapid evidence map suggested a gap in empirical research linking governance and conservation outcomes, particularly in relation to human well‐being. Indeed, this map highlighted the difficulty in studying the influence of governance on both biological and human well‐being outcomes, given those outcomes can take a long time to manifest and because governance operates in a multiscalar and complex manner with dynamic feedbacks across levels and time scales rather than linearly (Stark et al., 2022).

Until these knowledge gaps are addressed, and the available evidence base is improved upon, further attempts to use evidence synthesis approaches to assess the effects of environmental governance systems on conservation outcomes will remain difficult. Indeed, systematic reviews are part of a suite of decision tools, and researchers should consider other frameworks and tools, especially for urgent conservation questions (see, e.g., Bower et al., 2018; Rytwinski et al., 2021; Schwartz et al., 2018). However, to improve the current evidence base of governance and conservation effectiveness, we recommend that researchers, funders, international conservation organizations, and communities collaborate to facilitate interdisciplinary and transdisciplinary research. Such collaboration will lead to a more comprehensive understanding of the role of governance in conservation projects and ultimately result in more effective conservation efforts that benefit both biodiversity and human well‐being.

## Supporting information



Supporting Information

Supporting Information

Supporting Information

Supporting Information
