# Supplementary material for: Challenges in assessing the effects of environmental governance systems on conservation outcomes
Source: Conserv Biol. 2024 Oct 17;39(1):e14392. doi: 10.1111/cobi.14392 (PMC11780196; doi:10.1111/cobi.14392)
Supplement: Supplementary file 1 — Supporting Information [file COBI-39-e14392-s004.docx]

**Appendix S1. Search strategy and results**

Title: Challenges in assessing the effects of environmental governance systems on conservation outcomes

Authors: Raphael A. Ayambire, Trina Rytwinski, Jessica J. Taylor, Matthew W. Luizza, Matthew J Muir, Cynthia Cadet, Derek Armitage, Nathan J Bennett, Jeremy Brooks, Samantha H. Cheng, Jenny Martinez, Meenakshi Nagendran, Siri Öckerman, Shannon N. Rivera, Anne Savage, David S. Wilkie, Steven J. Cooke, Joseph R. Bennett

Description: This document provides a description of the search strategy and results of the literature searches. For each source, we provided full details on the search date(s), search strings used, search settings and restrictions, and subscriptions (if applicable), and the number of returns.

**Databases**

The search string was developed based on suggestions from the Advisory Team as described in the protocol of the review (CEBC, 2021).

The following bibliographic databases were searched in June or August 2021 using Carleton University’s institutional subscription:

1. ISI Web of Science Core Collection—multidisciplinary research topics including journals, books, proceedings, published data sets and patents
2. ProQuest Dissertation & Theses Global—collection of dissertations and theses from around the world, spanning from 1743 to present.
3. Scopus—abstract and citation database of peer-reviewed literature including journals, books, and conference proceedings.
4. Science.gov—US Federal Science

**Web of Science: Search Strategy #1**

Note: “Topic” search in Web of Science includes: title, abstract, keywords, keywords plus

Table 1. Metadata from Web of Science Search Strategy #1

| **Search string** | **Restrictions** | **Returns**  **[Date]** |
| --- | --- | --- |
| (TS=((Wildlife OR Fauna OR Animal$ OR Mammal$ OR ((Endangered OR Threatened OR vulnerable) NEAR/3 species) OR Elephant* OR Rhino* OR Antelope$ OR Gazell* OR Tiger$ OR Lion$ OR Panther$ OR Leopard$ OR Cheetah$ OR Ocelot$ OR Jaguar$ OR Pangolin$ OR Anteater$ OR "Ant eater$" OR Giraff* OR Okapi$ OR Primate$ OR Ape OR Apes OR Gorilla$ OR Chimpanzee$ OR Orangutan$ OR Gibbon$ OR Parrot$ OR Macaw$ OR Turtle$ OR Tortoise$ OR Cyca* OR Ivory OR Bushmeat$ OR Buffalo* OR flora) NOT (Rhinovirus* OR Rhinoplast*))) AND (TS= (“protected area$” OR (reserve$ NEAR/3 (natur* OR forest OR wildlife OR game OR private OR biosphere OR special)) OR “key biodiversity area$” OR “national park$” OR “wildlife sanctuar*” OR "wildlife refuge$" OR “wilderness area$” OR “marine protected area$” OR “MPA$” OR "private governance" OR "game farming" OR "wildlife ranching" OR "trophy hunting" OR “Community Resource Management Area$” OR “Wildlife Manage*” OR ("community based" NEAR/3 conservation) OR “CBC” OR ("community based" NEAR/3 management) OR “CBNRM” OR “community managed” OR ”community based governance” OR “collaborative management” OR (collaborative NEAR/3 governance) OR “collaborative management” OR “co-management” OR “comanagement” OR “environmental stewardship” OR “Wildlife Manage*” OR “wildlife governance” OR “community forest*” OR “Forest* Manage*” OR “Fisher* Manage*” OR “small scale” OR “Payment For Ecosystem Service$” OR “ecotourism” OR “Indigenous Peoples’ and Community Conserved Territories and Area$” OR “ICCA$” OR “Indigenous Protected Areas” OR “Locally Managed Marine Area$” OR "Indigenous" OR Aboriginal* OR "Native peoples" OR Tribal OR Tribe$ OR (conserv* NEAR/3 (governance OR area OR "community-led" OR private OR communit* OR status OR designation OR strateg* OR assessment$ OR policy OR policies OR significance OR action$ OR activit* OR manage* OR conservanc* OR covenant$ OR concession$ OR easement$ OR plan* OR priorit* OR decision)))) AND ((TS=("Population" OR "Relative size" OR Abundance$ OR Densit* OR Biomass OR Status OR Presence$ OR Distribution OR Range$ OR Occupanc* OR Detect* OR Recovery OR Progress OR Protect* OR Reproducti* OR Migration OR Behavior$ OR Behaviour$ OR "Genetic diversi*" OR Fecundity OR "Age structure" OR "Size structure" OR Recruitment OR "Biotic response" OR "Biological response" OR "Conservation target" OR Biodiversity OR "Ecological response" OR Impact OR Effectiveness OR Effective)) OR (TS = (“outcome$” OR “social outcome$” OR “social capital” OR “social impact$” OR “social justice” OR “socially just” OR “well-being” OR “wellbeing” OR awareness OR adoption OR "willingness to" OR welfare OR security OR livelihood OR job OR employment OR asset OR income OR decision-making OR govern* OR empower* OR participat* OR equity OR "human health" OR nutrition OR mortality OR disease OR consumption OR skill* OR degree OR train* OR literacy OR access OR "water clarity" OR "water quality" OR "clean water" OR "food security" OR vulnerability OR attitude* OR perception* OR "human capital" OR sanitation OR "building materials" OR housing OR fuel OR expenditure OR safety OR adapt* OR resilien* OR efficien* OR coproduction OR capability OR consensus OR integration))) | - All dates - **Web of Science Core Collection** - Advanced search - Topic field - All languages - All document types - Institution subscriptions:   - Science Citation Index Expanded (1900 - present)   - Social Sciences Citation Index (1956 - present)   - Arts & Humanities Citation Index (1975 - present)   - Conference Proceedings Citation Index - Science (1990 - present)   - Conference Proceedings Citation Index - Social Science and Humanities (1990 - present)   - Book Citation Index - Science & Social Science (2008 - present)   - Current Chemical Reactions (2008 - present)   - Index Chemicus (2008 - present)   - Part of the larger [Web of Science](https://library.carleton.ca/find/databases/web-knowledge). | **70,529** [August 25, 2021] |

**ProQuest Dissertations & Theses Global: Search Strategy #2**

Note: Command line advanced search selected to search within: title, abstract, keywords.

Table 2. Metadata from ProQuest Dissertations & Theses Global Search Strategy #2

| Search string | Restrictions | Returns  [Date] |
| --- | --- | --- |
| noft((Wildlife OR Fauna OR Animal? OR Mammal? OR ((Endangered OR Threatened OR vulnerable) NEAR/3 species) OR Elephant* OR Rhino* OR Antelope? OR Gazell* OR Tiger? OR Lion? OR Panther? OR Leopard? OR Cheetah? OR Ocelot? OR Jaguar? OR Pangolin? OR Anteater? OR "Ant eater?" OR Giraff* OR Okapi? OR Primate? OR Ape OR Apes OR Gorilla? OR Chimpanzee? OR Orangutan? OR Gibbon? OR Parrot? OR Macaw? OR Turtle? OR Tortoise? OR Cyca* OR Ivory OR Bushmeat? OR buffalo* OR flora) NOT (Rhinovirus* OR Rhinoplast*)) AND noft("protected area?" OR (reserve? NEAR/3 (natur* OR forest OR wildlife OR game OR private OR biosphere OR special)) OR "key biodiversity area?" OR "national park?" OR "wildlife sanctuar*" OR "wildlife refuge?" OR "wilderness area?" OR "marine protected area?" OR "MPA?" OR "private governance" OR "game farming" OR "wildlife ranching" OR "trophy hunting" OR "Community Resource Management Area?" OR "Wildlife Manage*" OR ("community based" NEAR/3 conservation) OR "CBC" OR ("community based" NEAR/3 management) OR "CBNRM" OR "community managed" OR "community based governance" OR "collaborative management" OR (collaborative NEAR/3 governance) OR "collaborative management" OR "co-management" OR "comanagement" OR "environmental stewardship" OR "Wildlife Manage*" OR "wildlife governance" OR "community forest*" OR "Forest* Manage*" OR "Fisher* Manage*" OR "small scale" OR "Payment For Ecosystem Service?" OR "ecotourism" OR "Indigenous Peoples’ and Community Conserved Territories and Area?" OR "ICCA?" OR "Indigenous Protected Areas" OR "Locally Managed Marine Area?" OR "Indigenous" OR Aboriginal* OR "Native peoples" OR Tribal OR Tribe? OR (conserv* NEAR/3 (governance OR area OR "community-led" OR private OR communit* OR status OR designation OR strateg* OR assessment? OR policy OR policies OR significance OR action? OR activit* OR manage* OR conservanc* OR covenant? OR concession? OR easement? OR plan* OR priorit* OR decision))) AND (noft("Population" OR "Relative size" OR Abundance? OR Densit* OR Biomass OR Status OR Presence? OR Distribution OR Range? OR Occupanc* OR Detect* OR Recovery OR Progress OR Protect* OR Reproducti* OR Migration OR Behavior? OR Behaviour? OR "Genetic diversi*" OR Fecundity OR "Age structure" OR "Size structure" OR Recruitment OR "Biotic response" OR "Biological response" OR "Conservation target" OR Biodiversity OR "Ecological response" OR Impact OR Effectiveness OR Effective) OR noft("outcome?" OR "social outcome?" OR "social capital" OR "social impact?" OR "social justice" OR "socially just" OR "well-being" OR "wellbeing" OR awareness OR adoption OR "willingness to" OR welfare OR security OR livelihood OR job OR employment OR asset OR income OR decision-making OR govern* OR empower* OR participat* OR equity OR "human health" OR nutrition OR mortality OR disease OR consumption OR skill* OR degree OR train* OR literacy OR access OR "water clarity" OR "water quality" OR "clean water" OR "food security" OR vulnerability OR attitude* OR perception* OR "human capital" OR sanitation OR "building materials" OR housing OR fuel OR expenditure OR safety OR adapt* OR resilien* OR efficien* OR coproduction OR capability OR consensus OR integration)) | - All dates - Dissertations & Theses Global - Master’s and doctoral dissertation - All languages - English only search terms - Institutional subscription   - Indexing 1743-present; Full text 1997-present   - PQDT Global includes theses from Great Britain and Ireland | **11,091** [August 25, 2021] |

**Scopus: Search Strategy #3**

Note: Advanced search selected to search within: title, abstract, and keywords.

Table 3. Metadata from Scopus Search Strategy #3

| Search string | Restrictions | Returns  [Date] |
| --- | --- | --- |
| (TITLE-ABS-KEY((Wildlife OR Fauna OR Animal OR Mammal OR ((Endangered OR Threatened OR vulnerable) W/3 species) OR Elephant* OR Rhino* OR Antelope OR Gazell* OR Tiger OR Lion OR Panther OR Leopard OR Cheetah OR Ocelot OR Jaguar OR Pangolin OR Anteater OR "Ant eater" OR Giraff* OR Okapi OR Primate OR Ape OR Gorilla OR Chimpanzee OR Orangutan OR Gibbon OR Parrot OR Macaw OR Turtle OR Tortoise OR Cyca* OR Ivory OR Bushmeat OR buffalo*OR flora) AND NOT (Rhinovirus* OR Rhinoplast*))) AND (TITLE-ABS-KEY(“protected area” OR (reserve W/3 (natur* OR forest OR wildlife OR game OR private OR biosphere OR special)) OR “key biodiversity area” OR “national park” OR “wildlife sanctuar*” OR "wildlife refuge" OR “wilderness area” OR “marine protected area” OR “MPA” OR "private governance" OR "game farming" OR "wildlife ranching" OR "trophy hunting" OR “Community Resource Management Area” OR “Wildlife Manage*” OR ("community based" W/3 conservation) OR “CBC” OR ("community based" W/3 management) OR “CBNRM” OR “community managed” OR ”community based governance” OR “collaborative management” OR (collaborative W/3 governance) OR “collaborative management” OR “co-management” OR “comanagement” OR “environmental stewardship” OR “Wildlife Manage*” OR “wildlife governance” OR “community forest*” OR “Forest* Manage*” OR “Fisher* Manage*” OR “small scale” OR “Payment For Ecosystem Service” OR “ecotourism” OR “Indigenous Peoples’ and Community Conserved Territories and Area” OR “ICCA” OR “Indigenous Protected Areas” OR “Locally Managed Marine Area” OR "Indigenous" OR Aboriginal* OR "Native peoples" OR Tribal OR Tribe OR (conserv* W/3 (governance OR area OR "community-led" OR private OR communit* OR status OR designation OR strateg* OR assessment$ OR policy OR policies OR significance OR action OR activit* OR manage* OR conservanc* OR covenant OR concession OR easement OR plan* OR priorit* OR decision)))) AND ((TITLE-ABS-KEY(Population OR "Relative size" OR Abundance OR Densit* OR Biomass OR Status OR Presence OR Distribution OR Range OR Occupanc* OR Detect* OR Recovery OR Progress OR Protect* OR Reproducti* OR Migration OR Behavior OR Behaviour OR "Genetic diversi*" OR Fecundity OR "Age structure" OR "Size structure" OR Recruitment OR "Biotic response" OR "Biological response" OR "Conservation target" OR Biodiversity OR "Ecological response" OR Impact OR Effectiveness OR Effective)) OR (TITLE-ABS-KEY(“outcome” OR “social outcome” OR “social capital” OR “social impact” OR “social justice” OR “socially just” OR “well-being” OR “wellbeing” OR awareness OR adoption OR "willingness to" OR welfare OR security OR livelihood OR job OR employment OR asset OR income OR decision-making OR govern* OR empower* OR participat* OR equity OR "human health" OR nutrition OR mortality OR disease OR consumption OR skill* OR degree OR train* OR literacy OR access OR "water clarity" OR "water quality" OR "clean water" OR "food security" OR vulnerability OR attitude* OR perception* OR "human capital" OR sanitation OR "building materials" OR housing OR fuel OR expenditure OR safety OR adapt* OR resilien* OR efficien* OR coproduction OR capability OR consensus OR integration))) | - All dates - Advanced search - All subject areas - All languages - All documents types - English only search terms | **116,566** [Aug 25, 2021] |

**Science.gov: Search Strategy #4**

### Available online: <https://www.science.gov/scigov/desktop/en/ostiblue/search.html>

Note: Advanced search searches Full Record, Title, Author, and Date Range. You cannot specify abstract, so full record was searched. Search is more limited than previous databases.

Table 4. Metadata from Science.gov Search Strategy #4

| Search string | Restrictions | Returns  [Date] |
| --- | --- | --- |
| fullrecord((Wildlife OR Fauna OR Animal? OR Mammal? OR "Endangered species" OR "Threatened species" OR "vulnerable species" OR Elephant* OR Rhino* OR Antelope? OR Gazell* OR Tiger? OR Lion? OR Panther? OR Leopard? OR Cheetah? OR Ocelot? OR Jaguar? OR Pangolin? OR Anteater? OR "Ant eater?" OR Giraff* OR Okapi? OR Primate? OR Ape OR Apes OR Gorilla? OR Chimpanzee? OR Orangutan? OR Gibbon? OR Parrot? OR Macaw? OR Turtle? OR Tortoise? OR Cyca* OR Ivory OR Bushmeat? OR flora) AND ("Population" OR "Relative size" OR Abundance? OR Densit* OR Biomass OR Status OR Presence? OR Distribution OR Range? OR Occupanc* OR Detect* OR Recovery OR Progress OR Protect* OR Reproducti* OR Migration OR Behavior? OR Behaviour? OR "Genetic diversi*" OR Fecundity OR "Age structure" OR "Size structure" OR Recruitment OR "Biotic response" OR "Biological response" OR "Conservation target" OR Biodiversity OR "Ecological response" OR Impact OR Effectiveness OR Effective OR “outcome?” OR “social outcome?” OR “social capital” OR “social impact?” OR “social justice” OR “socially just” OR “well-being” OR “wellbeing” OR awareness OR adoption OR "willingness to" OR welfare OR security OR livelihood OR job OR employment OR asset OR income OR decision-making OR govern* OR empower* OR participat* OR equity OR "human health" OR nutrition OR mortality OR disease OR consumption OR skill* OR degree OR train* OR literacy OR access OR "water clarity" OR "water quality" OR "clean water" OR "food security" OR vulnerability OR attitude* OR perception* OR "human capital" OR sanitation OR "building materials" OR housing OR fuel OR expenditure OR safety OR adapt* OR resilien* OR efficien* OR coproduction OR capability OR consensus OR integration)) | - All dates - Advanced search - All categories - Full record search terms - English only search terms - Accepted the additional records - Text records and public access included - Public access – peer-reviewed articles federally funded - Open access – no institutional subscription needed - NOTE: only Population and Outcome terms were used in the final string because the search function doesn’t allow NEAR that was used in the intervention, so search was kept broad to avoid missing articles | Text (577) Data (0) + Public Access (144)  [June 30, 2021] |

**Google Scholar**

We also conducted searches in Google Scholar in 2021 using three simplified search strings to search for additional commercially published and grey literature. The top 200 search results for each search string (sorted by relevance) were exported for screening in Excel.

*String 1:*

All of the words : Wildlife "community based"

At least one of the words: "Well being" Social Welfare Security Livelihood Health Population Abundance Density Effectiveness Effective Efficacy

*String 2:*

All of the words : Wildlife Governance

At least one of the words: "Well being" Social Welfare Security Livelihood Health Population Abundance Density Effectiveness Effective Efficacy

*String 3:*

All of the words : Wildlife Protected

At least one of the words: "Well being" Social Welfare Security Livelihood Health Population Abundance Density Effectiveness Effective Efficacy

**Additional sources**

We also hand-searched the bibliographies of 149 relevant reviews identified from the searches above to evaluate relevant titles that may not have been found using the search strategy. Additionally, we issued a call for evidence in 2021 to target sources of grey literature through relevant mailing lists (e.g., IUCN Green Criminology Specialist Group, Latin American Criminology Network, IUCN SULi), social media (e.g., CEBC twitter, Facebook, LinkedIn), and distribution to relevant networks and colleagues by Advisory Team.

**Literature Cited**

Canadian Centre for Evidence-Based Conservation (CEBC). (2021). What evidence exists on the effectiveness of community-based conservation and other governance types for meeting desired wildlife conservation and human well-being outcomes in Africa, Asia, and Latin America? A Systematic Map Protocol. *Zenodo*. https://doi.org/10.5281/zenodo.5670954
