# Supplementary material for: Challenges in assessing the effects of environmental governance systems on conservation outcomes
Source: Conserv Biol. 2024 Oct 17;39(1):e14392. doi: 10.1111/cobi.14392 (PMC11780196; doi:10.1111/cobi.14392)
Supplement: Supplementary file 2 — Supporting Information [file COBI-39-e14392-s001.docx]

**Appendix S2:** A list of eligible species and species groups.

Title: Challenges in assessing the effects of environmental governance systems on conservation outcomes

Authors: Raphael A. Ayambire, Trina Rytwinski, Jessica J. Taylor, Matthew W. Luizza, Matthew J Muir, Cynthia Cadet, Derek Armitage, Nathan J Bennett, Jeremy Brooks, Samantha H. Cheng, Jenny Martinez, Meenakshi Nagendran, Siri Öckerman, Shannon N. Rivera, Anne Savage, David S. Wilkie, Steven J. Cooke, Joseph R. Bennett

Description: This document provides the species and species groups native to Africa, Asia, or Latin America targeted by the United States Fish and Wildlife Service international grant making programs.

| **Species group** | **Order** | **Family name** | **Example species** |
| --- | --- | --- | --- |
| African & Asian elephants | Proboscidea | Elephantidae | *Loxodonta africana, Loxodonta cyclotis, Elephas maximus* |
| African & Asian rhinos | Perissodactyla | Rhinocerotidae | *Diceros bicornis, Ceratotherium simum, Rhinoceros unicornis, Rhinoceros sondaicus, Dicerorhinus sumatrensis* |
| Bovids | Cetartiodactyla | Bovidae | *Saiga tartaric, Oryx dammah, Addax nasomaculatus, Gazella dama* |
| Felids | Carnivora | Felidae | *Panthera tigris, Panthera leo, Panthera onca, Panthera uncia, Acinonyx jubatus, Panthera pardus, Leopardus pardalis* |
| Pangolins | Pholidota | Manidae | *Phataginus tricuspis, Phataginus tetradactyla, Smutsia gigantea, Smutsia temminckii, Manis pentadactyla, Manis crassicaudata, Manis javanica, Manis culionensis* |
| Giraffes & Okapi | Cetartiodactyla | Giraffidae | *Giraffa camelopardalis, Okapia johnstoni* |
| Parrots | Psittaciformes | Psittacoidea | *Psittacus erithacus, Ara macao* |
| Primates | Primates | Hominidae (non-human) | *Gorilla beringei, Gorilla gorilla, Pan troglodytes, Pan paniscus, Pongo abelii, Pongo pygmaeus, Pongo tapanuliensis* |
|  |  | Hylobatidae | *Symphalangus syndactylus, Hylobates agilis, Nomascus siki* |
|  |  | Callitrichidae | *Callithrix penicillata, Saguinus midas, Leontopithecus rosalia, Mico rondoni* |
|  |  | Cebidae | *Cebus olivaceus, Sapajus nigritus, Saimiri ustus* |
|  |  | Aotidae | *Aotus* spp. |
|  |  | Pitheciidae | *Callicebus coimbrai, Cacajao melanocephalus, Pithecia chrysocephala* |
|  |  | Atelidae | *Alouatta coibensis, Ateles fusciceps, Lagothrix lagotricha* |
| Turtles & Tortoises | Testudines | Cheloniidae | *Chelonia mydas, Caretta caretta, Lepidochelys kempii, Lepidochelys olivacea, Eretmochelys imbricata, Natator depressus* |
|  |  | Chelydridae | *Chelydra rossignonii, Chelydra acutirostris* |
|  |  | Dermochelyidae | *Dermochelys coriacea* |
|  |  | Dermatemydidae | *Dermatemys mawii* |
|  |  | Emydidae | *Terrapene coahuila* |
|  |  | Geoemydidae | *Cuora trifasciata*, *Cyclemys gemeli* |
|  |  | Kinosternidae | *Kinosternon angustipons, Staurotypus triporcatus, Claudius angustatus* |
|  |  | Pelomedusidae | *Pelomedusa galeata, Pelusios niger* |
|  |  | Platysternidae | *Platysternon megacephalum* |
|  |  | Podocnemididae | *Erymnochelys madagascariensis, Peltocephalus dumerilianus* |
|  |  | Testudinidae | *Chelonoidis chilensis,* *Homopus femoralis, Kinixys natalensis* |
|  |  | Trionychidae | *Chitra chitra*, *Lissemys punctata* |
| Cycads | Cycadales | Cycadaceae | *Cycas* spp. |
|  |  | Stangeriaceae | *Stangeria eriopus* |
|  |  | Zamiaceae | *Ceratozamia fuscoviridis*, *Encephalartos altensteinii* |
