# Supplementary material for: Challenges in assessing the effects of environmental governance systems on conservation outcomes
Source: Conserv Biol. 2024 Oct 17;39(1):e14392. doi: 10.1111/cobi.14392 (PMC11780196; doi:10.1111/cobi.14392)
Supplement: Supplementary file 3 — Supporting Information [file COBI-39-e14392-s002.docx]

# **Appendix S3. Rapid Evidence Map Screening Questions (i.e., inclusion criteria)**

Title: Challenges in assessing the effects of environmental governance systems on conservation outcomes

Authors: Raphael A. Ayambire, Trina Rytwinski, Jessica J. Taylor, Matthew W. Luizza, Matthew J Muir, Cynthia Cadet, Derek Armitage, Nathan J Bennett, Jeremy Brooks, Samantha H. Cheng, Jenny Martinez, Meenakshi Nagendran, Siri Öckerman, Shannon N. Rivera, Anne Savage, David S. Wilkie, Steven J. Cooke, Joseph R. Bennett

Description: This document provides the screening questionnaire used to determine the inclusion/exclusion of articles for the rapid evidence map.

**Screening Questionnaire**

1. Does this paper study the effect (i.e., evaluate qualitatively and/or quantitatively) of governance type on an eligible biological outcome(s)?
   - Must provide a clear link between governance and outcome (i.e., primary focus of the study was to test for a relationship between governance and outcome; explicitly stated in study hypothesis/prediction)
   - Must provide a direct link between governance type and outcome AND **include a comparator** (either before/after or control/impact or comparison between different types or levels of intervention but all types of governance need to be known)
   - See Table 1 of main text for types of environmental governance

Yes/No

1. Does this paper study the effect (i.e., evaluate qualitatively and/or quantitatively) of governance type on an eligible human well-being outcome(s) (HWB)?
   1. Must provide a clear link between governance and outcome (i.e., primary focus of the study was to test for a relationship between governance and outcome; explicitly stated in study hypothesis/prediction)
   2. Must provide a direct link between governance type and outcome AND **include a comparator** (either before/after or control/impact or comparison between different types or levels of intervention but all types of governance need to be known)
   3. See Table 1 of main text for types of environmental governance

Yes/No

If NO to 1 AND 2, Indicate N/A for 3,4,5

1. Is this a primary study?

Yes/No

If NO to 3, Indicate N/A for 4,5

1. Is the species or species group native to Africa, Asia, or Latin America and targeted by the United States Fish and Wildlife Service international activities AND/OR pertain to a ‘local community’ (i.e., group of actors with access to, using, benefiting from, or affecting a specific eligible species and associated with a conservation governance type) in the three eligible regions of Africa, Asia, and Latin America (see Appendix S2 for full list of eligible species/species groups)?

Yes/No/Unclear

If NO to 4, Indicate N/A for 5

1. Does this paper provide information on governance principles within a conservation governance type(s) (i.e., provides sufficient information to identify the presence/absence of governance principles)?

Yes/No

If YES, use the free-text note to list any evidence. See Table 2 of main text for examples.

1. General Notes (Free text): Use this column to explain the answers for Qs 1-5

ONLY use UNCLEAR option for Question 4 (see below).

## **Gap Map matrix**

|  | Direct link with a conservation governance type | Sufficient information to identify presence/absence of governance principles |  |
| --- | --- | --- | --- |
|  |  |  |  |
| Biological outcome(s) | Yes Q#1 (as well as #3, 4) but No Q#2 | Yes Q#5 (as well as #1, 3, 4) but No Q#2 |  |
| HWB outcome(s) | Yes Q#2 (as well as #3, 4) but No Q#1 | Yes Q#5 (as well as #2, 3, 4) but No Q#1 |  |
| Biological & HWB | Yes Q#1&2 (as well as #3, 4) | Yes Q#5 (as well as #1, 2, 3 & 4) |  |

## **Further Screening details:**

### **Outcomes**

Biological outcomes, on relevant species or species groups (listed in Appendix S2), include 11 outcome metrics which are outlined in Table 1 below. Human well-being outcomes encompass four domains, provided below in Table 2 with explanation, focal areas, and example indicators. We include studies that measure the effects or change on biological and/or human well-being outcomes either qualitatively and/or quantitatively; however, there needs to be a direct link between eligible outcomes and conservation governance type(s) to be included. We exclude studies reporting on ecological or habitat-related outcomes (e.g., species diversity, habitat quality/stability), or on evolutionary phenomena and processes. Note that components of Ecosystem Health must directly link to a human population (e.g., subsistence, drinking water quality) to be included.

Table 1. Definition and eligible outcome metrics for biological outcomes on target species.

|  | **Definition** | **Eligible outcome metrics** |
| --- | --- | --- |
| **Biological outcomes** | Outcomes focusing on change in populations of individuals or populations within species. | Abundance/density (e.g., number of individuals per unit area, presence/absence) |
|  |  | Biomass (e.g., animal or plant dry mass per unit area) |
|  |  | Age/size structure (e.g., length/weight/age distributions of individuals in a population) |
|  |  | Reproduction (e.g., fecundity, # of offspring/reproductive individuals), |
|  |  | Recruitment (e.g., # of individuals that have joined a population over some time period) |
|  |  | Behavior (e.g., time spent feeding/hiding, hunting behaviour)  *Note – if diet composition studies provide a direct link to hunting behavior they can be included. If only providing baseline diet information, they are excluded. |
|  |  | Species range/spatial extent (e.g., expansion or contraction of a species range limit and/or extent) |
|  |  | Dispersal (e.g., migration and/or connectivity patterns) |
|  |  | Connectivity (e.g., measures of a degree to which populations are interacting) |
|  |  | Body condition (e.g., incidence of disease, injury by traps)  *Note – if health studies provide a direct link to body condition or the animal’s nutrient status/health they can be included. If only providing baseline health information, they are excluded. |
|  |  | Mortality (e.g., human-induced, disease) |
|  |  | Adaptability (e.g., genetic diversity) |

Table 2. Human well-being (HWB) outcome domains, focal areas, and their explanations, with example indicators.

| **HWB Domain/ Explanation** | **HWB Focal Area/ Explanation** | **Example indicators** |
| --- | --- | --- |
| **Economic:**  Sources/ activities and levels of economic resources. | **Employment & Livelihoods:**  Presence of secure and diverse sources of employment and livelihoods. | Employment rates; unemployment trends/rates; livelihood diversity & availability; ability to meet costs and obtain necessary permits; access to markets; sense of economic security; resource-based livelihoods (extractive and non-extractive); non-resource-based livelihoods; local business activity; productivity; opportunity; seasonality |
|  | **Income & Assets:**  Levels of economic and material wealth in the form of diverse sources of income, and accrual of assets. | Income; wages; after-tax income rates; debt rates; income per unit effort; affordability; savings; assets; land, livestock capital; household material assets index; possessions; material living standards; informal economy characteristics (e.g., barter and trade systems) |
| **Health:**  Affordability and accessibility of services and resources that promote physical and mental health. | **Food Security:**  Affordability and accessibility of healthy and reliable food source(s). | Household or child food security index; affordability and accessibility of healthful food; food availability and variety; local fisheries and agricultural harvests; toxin levels in local shellfish harvest areas; nutrition |
|  | **Healthcare & Basic Services:**  Affordability and accessibility of healthcare facilities and medical practitioners, and access to basic services. | Infant/under 5/adult mortality rate; disease rate; life expectancy; birth rate; access to health care services; incidence of depression and anxiety; mental illness; addiction rates; access to addiction support services; mental health outreach services; access to clean drinking water; transit services; sanitation and recycling services; public utilities and transit; public expenditures; availability/access to electricity |
|  | **Ecosystem Health:**  The status and productivity of diverse ecosystem services including aspects related to provisioning (e.g., food and medicinal resources), regulating (e.g., climate regulation), and cultural (e.g., cultural identity, aesthetic values). | Fisheries catch; mangrove biomass; recreational services/areas; annual value of tourism; productivity; pollination; land use intensity; levels of grazing, harvest, planting intensity; air, soil & water quality; invasive species; habitat fragmentation & degradation; restored habitats |
| **Social Considerations:**  Strength of social relations, and levels of education and resilience. | **Social Relations:**  Presence of positively perceived interactions, levels of trust, and the ability to resolve conflicts, between individuals and partner organizations. | Sense of community; community spaces; trust in neighbors; conflicts between groups; social conflicts; perceptions of discrimination; quality of cooperation and cohesiveness of the community |
|  | **Education:**  Affordability and accessibility of educational infrastructure and knowledge, including preserving local ecological/ traditional knowledge. | School enrolment rates; literacy rates; school attendance & achievement; cost/ease of access to education and specialized training; access to information; environmental education, awareness of threats to environment; awareness of conservation actions; knowledge of sustainable practices; skills and capacity gained; trainings conducted and access to tools or technology |
|  | **Safety and security:**  Actual and perceived levels of safety, freedom from violence and conflict, and fair treatment from authorities. | Crime rates; crime severity; acts of violence; occurrence of theft; livestock theft; sense of personal security; sense of community security; sense of safety walking after dark; unjustified arrest; abuse of power by authorities |
| **Culture & Cognition:**  Recognition of social, political and cultural identities or products of a particular group of people. Presence of positive individual/ community emotions. | **Identity:**  Recognition of social, political and cultural identities as perceived by individuals / the community. | Sense of control; sense of identity; ethnic identity; place attachment; sense of place; knowledge of the natural environment; sense of connection to livelihood; self-definition (at individual or community levels); use, preservation, and transfer of traditional ecological knowledge and place names; participation in cultural practices; continuity of practices and customs; traditional use of resources; traditional management activities; cultural festivals and event |
|  | **Emotions:**  Presence of positive emotions at the community/ individual level including pride, relief, hope and optimism for the intervention. | Confidence in the future; feelings of optimism for the future; feeling of happiness; job satisfaction; overall life satisfaction |
|  | **Autonomy:**  Opportunities to make choices and act in manner valued (culturally) by individuals/ the community. | Capacity to self-organize; ability to control one’s own life; financial independence; sense of control and power; sense of agency; ability to pursue what one values doing and being; ability to participate in cultural practices and cultivate traditional foods and medicines |

### **Study Type**

We are only including primary studies. This includes studies collecting primary data investigating the effects of a conservation governance type on a biological and/or human well-being outcome(s) on this specific topic. We are excluding reviews, policy discussions, commentaries, or theoretical modeling studies.

### **Population**

This includes wild animal and plant species and species groups outlined below in Appendix S2. Example species listed, in most cases, are just that, examples, and eligibility will be based on at the Family level (i.e., defer to Family when screening). If the species belongs to one of the eligible families AND the species is native to Africa, Asia, or Latin America (can search for species distribution data using the IUCN Red List <http://www.iucnredlist.org/>) **= Yes**.

If the species is not native to Africa, Asia, or Latin America = **No.** For the purpose of this exercise, Latin America will include all of the Americas south of the United States (i.e., South America, Caribbean, Central America including Mexico). Include all of Russia.

If the study does not focus on a species or species group specifically (e.g., no species are mentioned or only a general list of species in the area is provided; examples provided in next paragraph), or it is unclear whether it is explicitly targeting a relevant species or species group, AND the study takes place in Africa, Asia, or Latin America = **UNCLEAR**

For example, in cases where the study is evaluating the effectiveness of CBNRM programs (e.g., Conservation Conservancy, Community-based harvesting programs), but there is either (1) no mention of the target species or species group being studied, and no indication of which species are found in the area (but it is possible that relevant species could be there), or (2) the study provides a list of species found in the area (and one or more are relevant to this map) but it does not state if all or some of those species were the target of the study.

With respect to HWB outcomes: HWB Impacts measured must be on an eligible local community = Indigenous Peoples and local communities associated with a specific conservation governance intervention (on a relevant species/species group) located within Africa, Asia, and Latin America.
